# Synergistic action of SPI-1 gene expression in *Salmonella enterica* serovar typhimurium through transcriptional crosstalk with the flagellar system

**DOI:** 10.1186/s12866-019-1583-7

**Published:** 2019-09-05

**Authors:** Selwan Hamed, Xiaoyi Wang, Riham M. Shawky, Mohamed Emara, Philip D. Aldridge, Christopher V. Rao

**Affiliations:** 1 Department of Chemical and Biomolecular Engineering, University of Illinois at Urbana-Champaign, 600 S. Mathews Ave, Urbana, IL 61801 USA; 2 Department of Microbiology and Immunology, Faculty of Pharmacy, Helwan University − Ain Helwan, Helwan, 11795 Egypt; 3 Institute of Cell & Molecular Biosciences, Faculty Medical Sciences, Newcastle University, Newcastle upon Tyne, UK

**Keywords:** Salmonella, SPI-1, Flagella, Bistability, Gene regulation, Crosstalk, Acetate

## Abstract

**Background:**

*Salmonella enterica* serovar Typhimurium is a common food-borne pathogen. *S. enterica* uses a type III secretion system encoded within *Salmonella* pathogenicity island 1 (SPI-1) to invade intestinal epithelial cells. A complex network of interacting transcription factors regulates SPI-1 gene expression. In addition, SPI-1 gene expression is coupled to flagellar gene expression. Both SPI-1 and flagellar gene expression are bistable, with co-existing populations of cells expressing and not expressing these genes. Previous work demonstrated that nutrients could be used to tune the fraction of cells expressing the flagellar genes. In the present study, we tested whether nutrients could also tune the fraction of cells expressing the SPI-1 genes through transcriptional crosstalk with the flagellar genes.

**Results:**

Nutrients alone were not found to induce SPI-1 gene expression. However, when the cells were also grown in the presence of acetate, the concentration of nutrients in the growth medium was able to tune the fraction of cells expressing the SPI-1 genes. During growth in nutrient-poor medium, acetate alone was unable to induce SPI-1 gene expression. These results demonstrate that acetate and nutrients synergistically activate SPI-1 gene expression. The response to acetate was governed by the BarA/SirA two-component system and the response to nutrients was governed by transcriptional crosstalk with the flagella system, specifically through the action of the flagellar regulator FliZ.

**Conclusions:**

Acetate and nutrients are capable of synergistically activating SPI-1 gene expression. In addition, these signals were found to tune the fraction of cells expressing the SPI-1 genes. The governing mechanism involves transcriptional crosstalk with the flagellar gene network. Collectively, these results further our understanding of SPI-1 gene regulation and provide the basis for future studies investigating this complex regulatory mechanism.

**Electronic supplementary material:**

The online version of this article (10.1186/s12866-019-1583-7) contains supplementary material, which is available to authorized users.

## Background

*Salmonella enterica* serovar Typhimurium is a common food-borne pathogen. It is responsible for a wide range of diseases in humans, ranging from self-limiting gastroenteritis to life-threatening systemic infections [1, 2]. *S. enterica* uses a type III secretion system encoded within *Salmonella* pathogenicity island 1 (SPI-1) to invade intestinal epithelial cells [3]. A complex network of interacting transcription factors regulates the expression of the SPI-1 genes [4]. HilA is the master regulator of SPI-1 gene expression, because it activates the expression of structural genes encoding the type III section system. HilA expression, in turn, is regulated by three transcription factors: HilC, HilD, and RtsA, which positively regulate their own expression and that of each other [5]. Among these three factors, HilD is the main regulator of HilA expression – many of the signals inducing SPI-1 gene expression do so by altering the expression or activity of HilD. HilC and RtsA, on the other hand, appear to simply amplify HilA expression [5, 6].

**Fig. 1:**
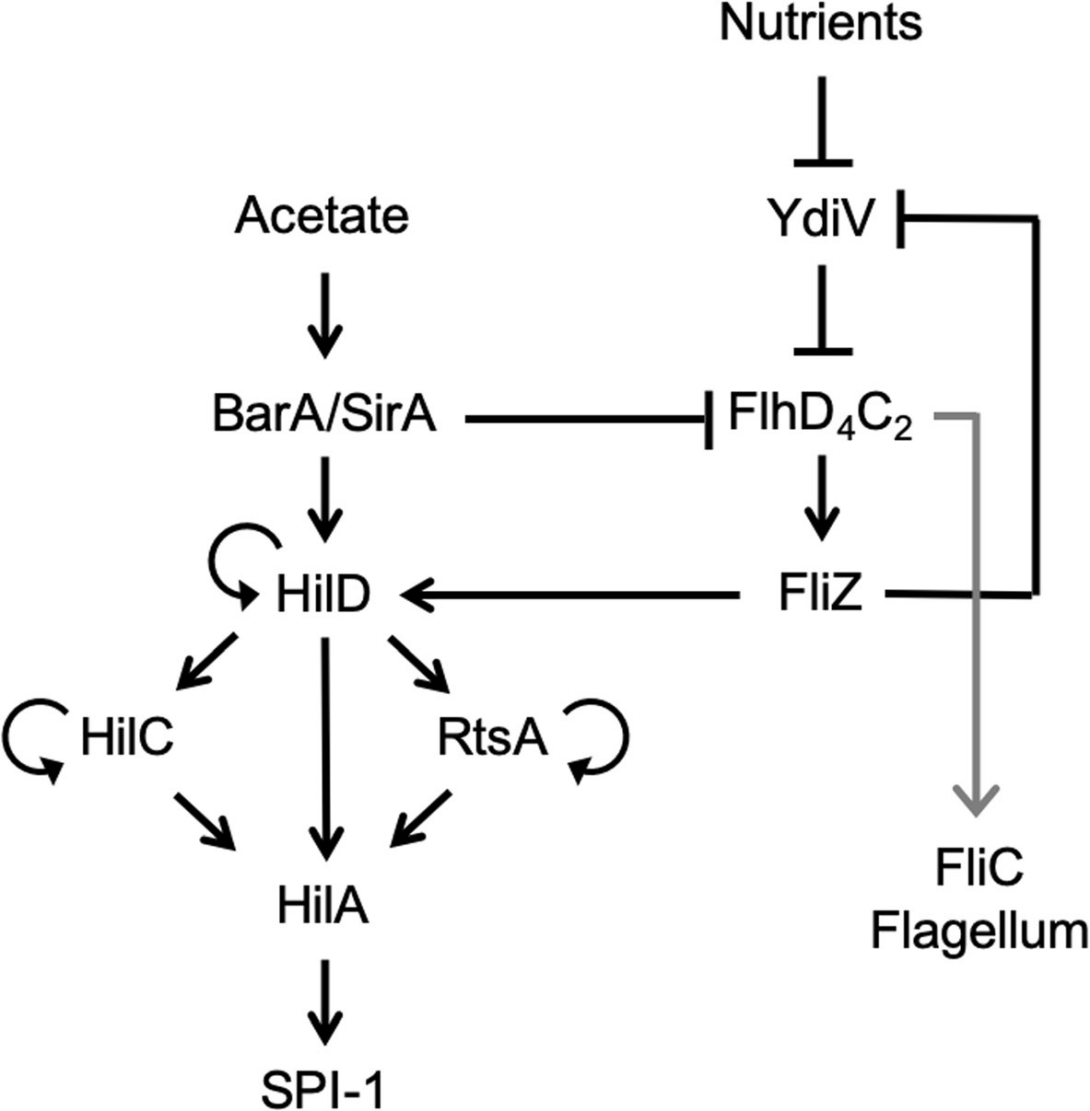
Regulatory network controlling SP1–1 and flagellar gene expression. Only the key regulators and relevant proteins in the context of the present study are shown. See main text for details

SPI-1 gene expression is also coupled with flagellar gene expression [7–16]. Briefly, the flagellum is a rotary motor that enables bacteria to swim in liquids and to swarm over surfaces [17]. Similar to the SPI-1 gene network, multiple transcription factors also regulate the expression of flagellar genes [18]. The master regulator of flagellar gene expression is FlhD_4_C_2_. In addition to activating the expression of the flagellar structural genes, it also activates the expression of FliZ, which indirectly regulates FlhD_4_C_2_ by repressing the expression of YdiV (also known as RflP) [19, 20]. YdiV binds to FlhD_4_C_2_ and prevents it from activating class 2 flagellar promoters. It also promotes the degradation of FlhD_4_C_2_ [20, 21]. In addition to regulating flagellar gene expression, FliZ regulates SPI-1 gene expression by activating HilD via an unknown post-translational mechanism [14]. Moreover, HilD can activate the transcription of *flhDC* [7], whereas RtsB, which is encoded in the same operon as RtsA, can repress the transcription of *flhDC* [10]. Collectively, these regulatory mechanisms couple the expression of the SPI-1 and flagellar genes (Fig. 1).

Both the flagellar and SPI-1 genes exhibit bistable expression patterns (cf. [6, 22–33]): within a population of cells, some cells will express these genes and others will not. In other words, only a subpopulation of cells will be motile or invasive. Previous work found that the nutritional content of the growth medium controls the fraction of cells expressing the flagellar genes in a bistable manner [30]. In addition, the mechanism governing this bistable response was determined. In the present work, we investigated whether the nutrients also tune the fraction of cells expressing the SPI-1 genes. In addition, we explored whether transcriptional crosstalk with the flagellar genes also governs the response of the SPI-1 genes to nutrients.

## Results

### Synergistic activation of SPI-1 gene expression by acetate and yeast extract

Previous work has shown that flagellar gene expression is bistable in *S. enterica*, and that the fraction of motile cells is determined by the nutrient concentration in the growth medium [30]. In these experiments, cells were grown in Vogel-Bonner medium E containing 0.2% glucose (hereafter referred to as VB medium) and varying concentrations of yeast extract, which was used to tune the nutrient concentration. Increasing the amount of yeast extract in the growth medium was found to increase the fraction of cells expressing the flagellar genes in a bistable manner. Because the expression of the SPI-1 genes in *S. enterica* is coupled to the expression of the flagellar genes, we sought to determine whether nutrients would also tune the expression of the SPI-1 genes. To measure gene expression, we employed single-copy, chromosomally integrated transcriptional fusions of the *hilA* promoter to the green fluorescent protein (GFP). Flow cytometry was used to measure fluorescence at single-cell resolution to account for the possibly bistable expression of the SPI-1 genes. When we grew the cells in VB medium at vary concentrations of yeast extract, expression from the *hilA* promoter was weak. In particular, yeast extract had only a small effect on expression, increasing it 2.8 fold (Additional file 1: Fig. S1 and S2). These results were initially surprising as the addition of yeast extract strongly induce flagellar gene expression under identical growth conditions.

**Fig. 2:**
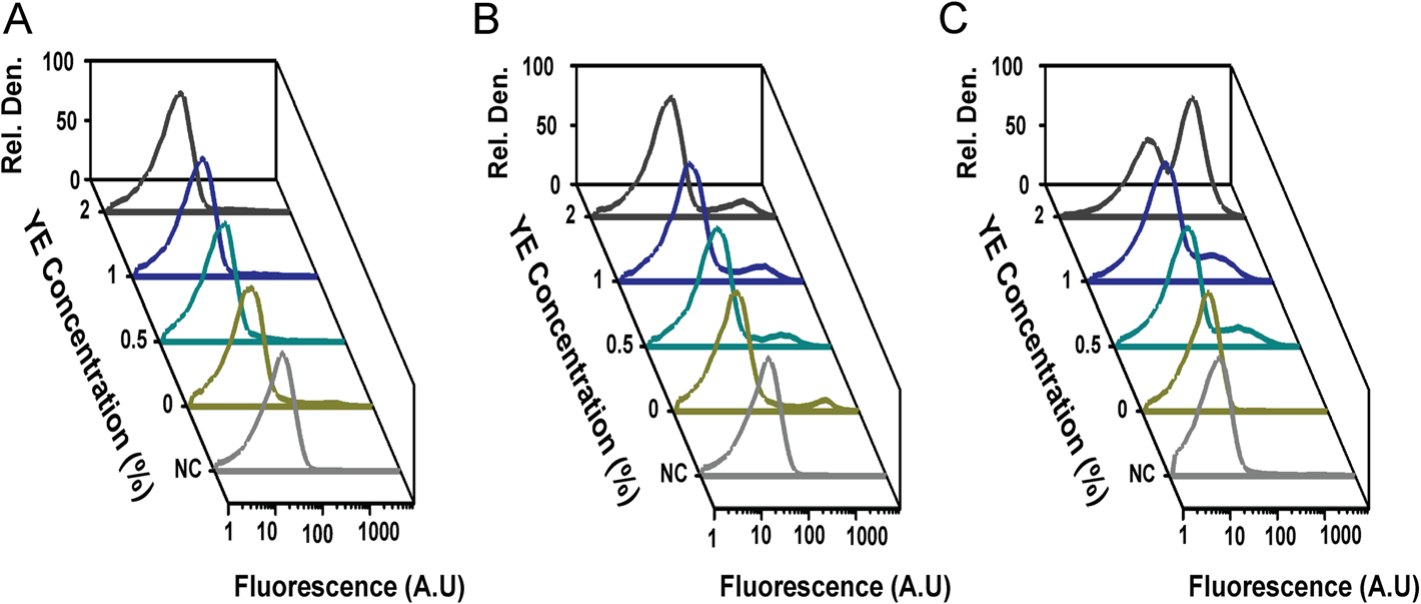
Acetate and yeast extract (YE) synergistically induce expression from the *hilA* promoter during growth in TB medium. Expression from *hilA* promoter was determined using single-copy transcriptional fusions to GFP as determined using flow cytometry. *hilA* promoter activity in wild-type cells during: growth in TB medium (**a**); growth in TB medium containing 1% NaCl (**b**); and growth in TB medium containing 10 mM sodium acetate (**c**) at various concentrations of yeast extract. Negative control (NC) is the measured fluorescence of wild-type cells not containing the *gfp* gene during growth in TB medium. Analysis of data is provided in Fig. 3

SPI-1 gene expression is induced during growth in lysogeny broth (LB), which consists of tryptone, yeast extract, and salt [34, 35]. Therefore, we first tested whether growth in tryptone broth, lacking salt (hereafter referred to as TB medium) and containing varying concentrations of yeast extract, would induce SPI-1 gene expression. As a control, we first measured flagellar gene expression in this growth medium using a single-copy transcriptional fusion of the *fliC* promoter to the fluorescent protein Venus. During growth in VB and TB media, the addition of yeast extract increased expression from the *fliC* promoter (Additional file 1: Fig. S3 and S4). The key difference is that expression was bistable, with co-existing population of *fliC*-expressing (motile) and non-expressing (non-motile) cells, during growth in VB medium, as previously reported [30], but not in TB medium, where expression was monostable. In addition, yeast extract yielded a 54-fold increase in the mean level of *fliC* expression during growth in VB medium as compared to a 4-fold increase during growth in TB medium. The difference is due to the higher level of expression during growth in TB medium in the absence of yeast extract. The mean level of expression is similar when the media contain 2% yeast extract. When *hilA* expression was measured during growth in TB medium, we again observed a small, 2.5-fold increase in the mean level of expression (Fig. 2a). Once again, yeast extract had only a small effect on *hilA* promoter activity.

**Fig. 3:**
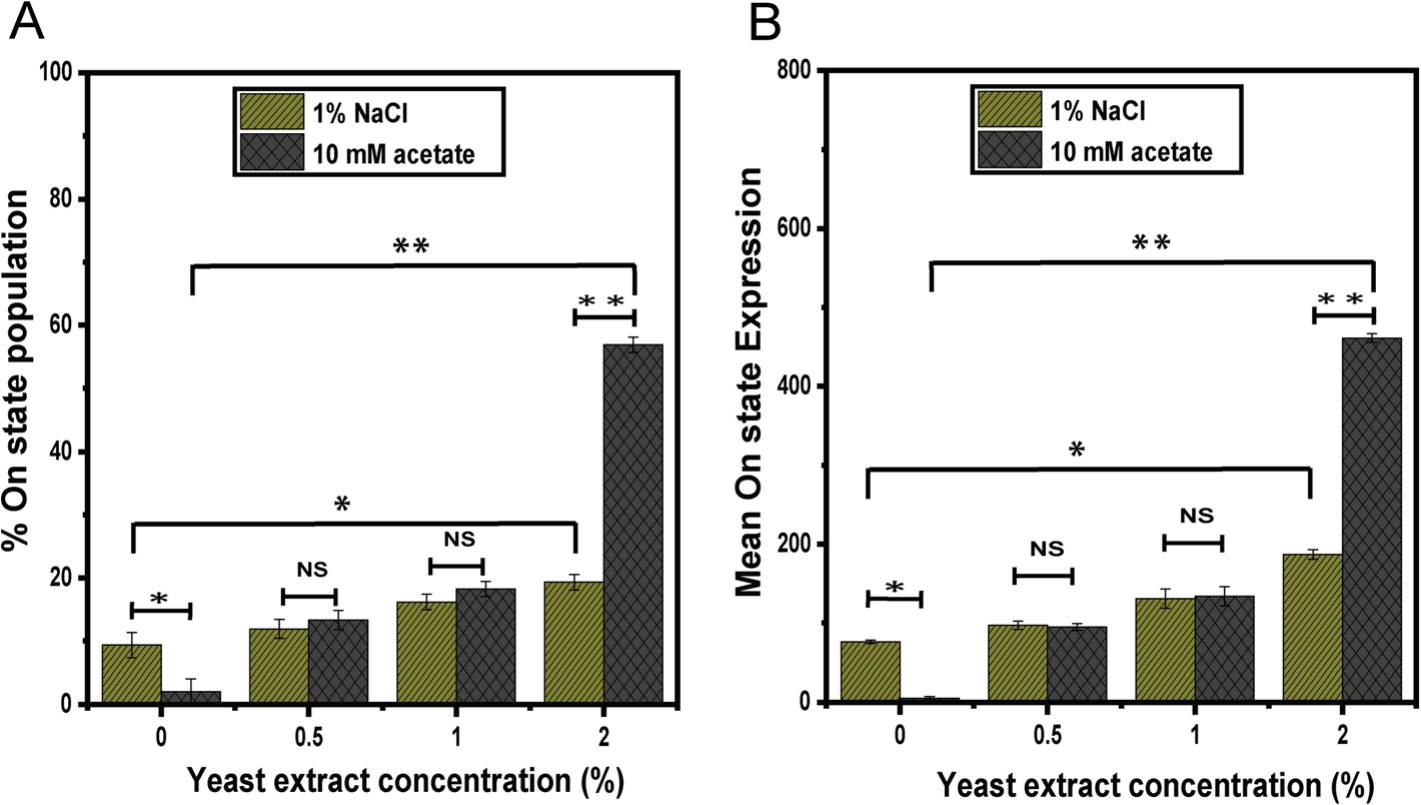
Fraction of cells expressing *hilA* (on state) during growth in TB medium containing acetate or yeast extract (YE) (**a**). Mean expression of *hilA* promoter for cells in the on state (**b**). Expression from *hilA* promoter was determined using single-copy transcriptional fusions to GFP as determined using flow cytometry. Fraction of cells in the on state was determined relative to the negative control (100% in the off state), which consisted of the measured fluorescence of wild-type cells not containing the *gfp* gene during growth in TB medium Representative data are provided in Fig. 2. Error bars denote the standard deviation from three biological replicates

Both salt and acetate are known to induce SPI-1 gene expression [35–37]. Therefore, we tested whether the addition of 1% sodium chloride or 10 mM sodium acetate would affect SPI-1 gene expression under the growth conditions explored above. When we grew cells in TB containing 1% salt and varying concentrations of yeast extract, we observed a small (9%) population of cells where the *hilA* promoter was active (Fig. 2b). However, in the majority of cells, the *hilA* promoter was inactive. Moreover, the addition of yeast extract yielded a small, 2-fold increase in the fraction of cells expressing the SPI-1 genes, as inferred from *hilA* promoter activity (Fig. 3a). Because the effect was minor, we did not explore further.

When acetate was added to the growth medium, however, yeast extract yielded a large, 950-fold increase the fraction of cells expressing the SPI-1 genes (on state) and a 230-fold increase in the mean level of expression within the on-state population (Figs. 2c and 3). Expression was bistable, with co-existing population of cells expressing and not expressing the SPI-1 genes. Interestingly, acetate alone did not induce SPI-1 gene expression in the absence of yeast extract. Likewise, yeast extract alone was unable to induce SPI-1 gene expression in the absence of acetate. Collectively, these results demonstrate that acetate and yeast synergistically activate SPI-1 gene expression.

The same behavior was not observed when the base medium was VB (Additional file 1: Fig. S5 and S6). In particular, acetate yielded a small, less than 2-fold, increase in *hilA* expression in the presence of yeast extract. Expression was also monostable. These results suggest that growth in VB medium does not induce SPI-1 gene expression. It is possible that components in this medium repress SPI-1 gene expression. Therefore, we focused on TB medium for the remaining study.

As a control, we also performed similar experiments using single-copy transcriptional fusions of the *hilD* promoter to Venus. During growth in TB medium (Additional file 1: Fig. S7 and S8), yeast extract had a small (< 2-fold) effect on *hilD* promoter activity. When the cells were also grown in the presence of acetate, yeast extract induces bistable expression from the *hilD* promoter, similar to what is observed with the *hilA* promoter. These results suggest that acetate and yeast extract are inducing the *hilD* promoter, which sits atop of the SPI-1 regulatory network. However, the effects are far less pronounced effect as compared to *hilA* expression, as mean expression increased only 3.5-fold. One possibility is that HilC and RtsA amplify the response.

### Response to acetate is mediated through the BarA/SirA two-component system

**Fig. 4:**
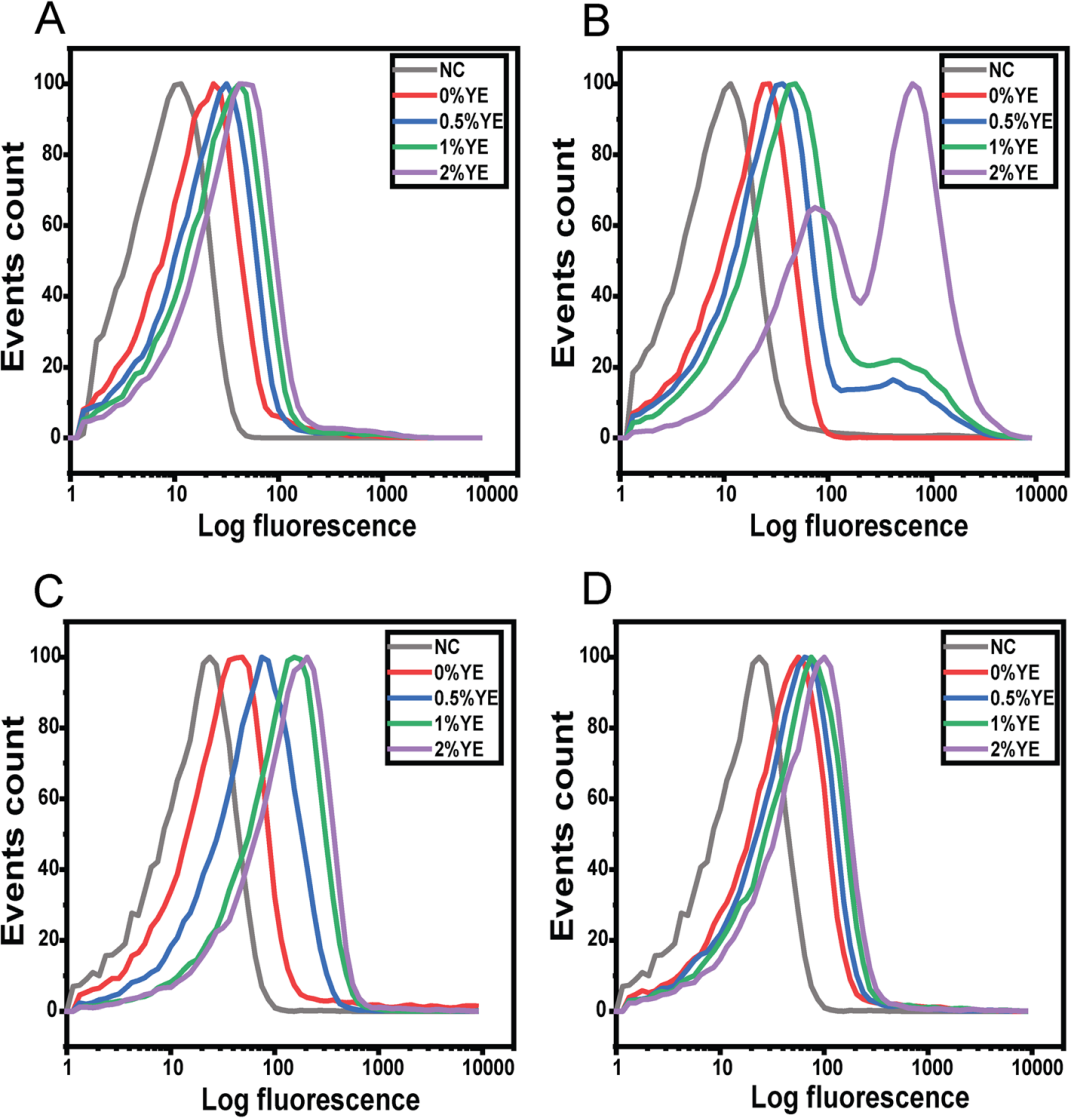
Response of the *hilA* promoter to acetate is regulated by BarA/SirA two-component signal transduction system. Expression from *hilA* promoter was determined using single-copy transcriptional fusions to GFP as determined using flow cytometry. **a.** *hilA* promoter activity in the wild type during growth in TB medium at various concentrations of yeast extract (YE). **b.** *hilA* promoter activity in the wild type during growth in TB medium containing 10 mM acetate at various concentrations of yeast extract (YE). **c**. *hilA* promoter activity in a Δ*sirA* mutant during growth in TB medium at various concentrations of yeast extract (YE). **d**. *hilA* promoter activity in a Δ*sirA* mutant during growth in TB medium containing 10 mM acetate at various concentrations of yeast extract (YE). Negative control (NC) is the measured fluorescence of wild-type cells not containing the *gfp* gene during growth in TB medium. Panels A and B are shown for comparative purposes and are the same results as shown in Fig. 2. Analysis of data is provided in Additional file 1: Fig. S9

The ability of acetate to induce SPI-1 gene expression is regulated by the BarA/SirA two-component signal transduction system [36]. Therefore, we tested the ability of acetate and yeast extract to induce SPI-1 gene expression in a Δ*sirA* mutant during growth in TB medium (Fig. 4). In this case, yeast extract and acetate no longer induce bistable expression from the *hilA* promoter. Rather, expression was monostable. In addition, the degree of activation by acetate and yeast extract was significantly less than the wild type: less than a 2-fold increase in mean expression for the Δ*sirA* mutant versus a 22-fold increase for the wild type.

### Response to yeast extract is mediated through FliZ

Yeast extract is known to induce flagellar gene expression by repressing the expressing of YdiV [38]. YdiV binds to FlhD_4_C_2_, the master regulator of flagellar gene expression, and prevents it from activating the class 2 flagellar promoters [21, 38]. In addition, it promotes the degradation of FlhD_4_C_2_ through ClpXP [21]. Thus, yeast extract induces flagellar gene expression by repressing the expression of a negative regulator of FlhD_4_C_2_. In addition, FliZ, whose expression is induced by FlhD_4_C_2_, represses *ydiV* transcription [20]. This double negative-feedback loop generates the bistable response of flagellar gene expression to yeast extract [30]. In addition to being a regulator of flagellar gene expression, FliZ also induces SPI-1 gene expression by increasing HilD expression [14], thus, providing one mechanism for coupling the expression of these two systems.

**Fig. 5:**
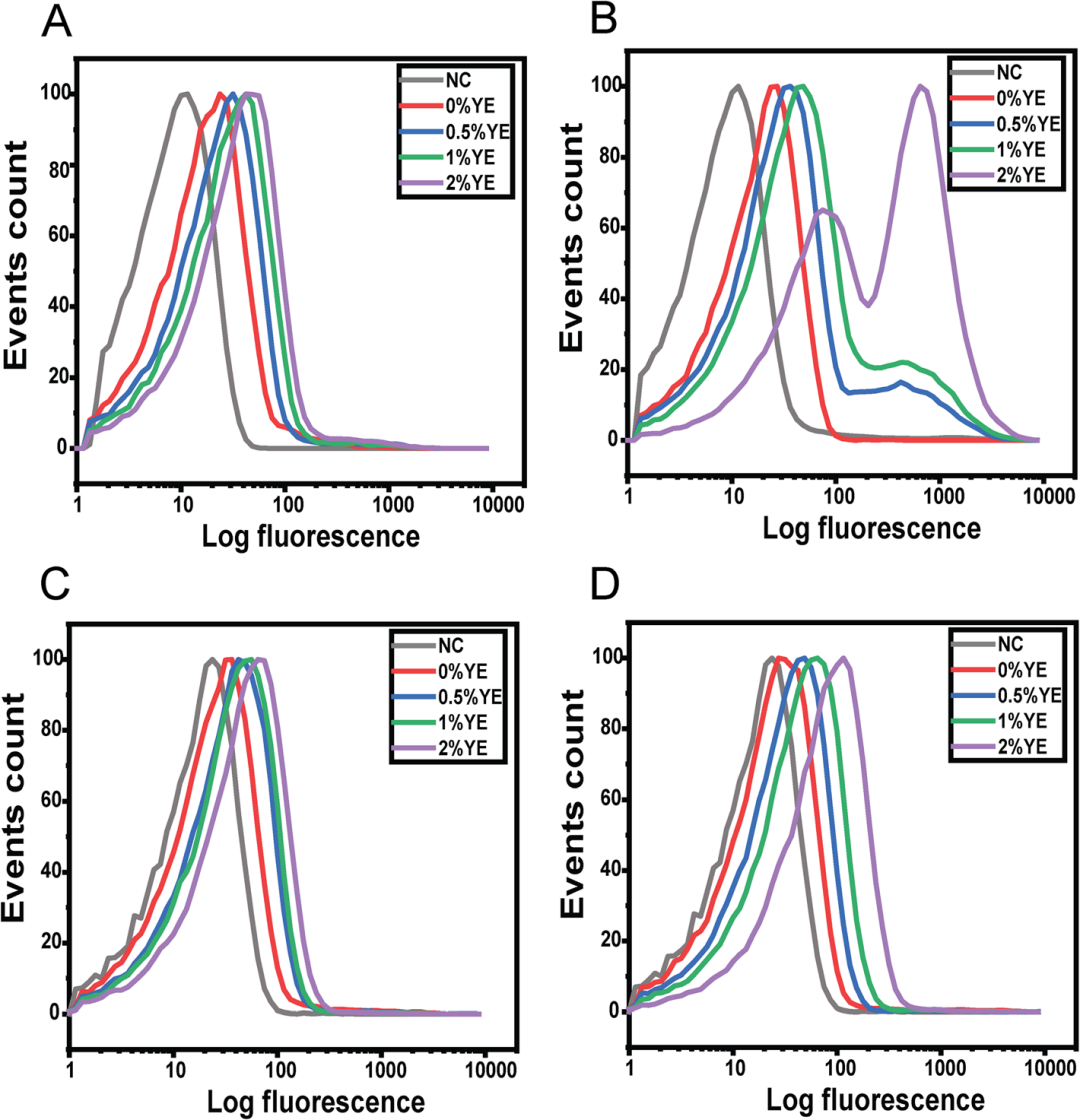
Response of the *hilA* promoter to yeast extract is due to transcriptional crosstalk with the flagellar system. Expression from *hilA* promoter was determined using single-copy transcriptional fusions to GFP as determined using flow cytometry. **a.** *hilA* promoter activity in the wild type during growth in TB medium at various concentrations of yeast extract (YE). **b.** *hilA* promoter activity in the wild type during growth in TB medium containing 10 mM acetate at various concentrations of yeast extract (YE). **c**. *hilA* promoter activity in a Δ*fliZ* mutant during growth in TB medium at various concentrations of yeast extract (YE). **d**. *hilA* promoter activity in a Δ*fliZ* mutant during growth in TB medium containing 10 mM acetate at various concentrations of yeast extract (YE). Negative control (NC) is the measured fluorescence of wild-type cells not containing the *gfp* gene during growth in TB medium. Panels A and B are shown for comparative purposes and are the same results as shown in Fig. 2. Analysis of data is provided in Additional file 1: Fig. S10

We hypothesized that induction of SPI-1 gene expression by yeast extract in the presence of acetate is mediated through transcriptional crosstalk with the flagellar system. To test this hypothesis, we measured *hilA* promoter activity in a Δ*fliZ* mutant during growth in TB medium (Fig. 5). In the presence of acetate, yeast extract yielded a 2.5-fold increase in mean *hilA* expression in a Δ*fliZ* mutant versus a 22-fold increase in the wild type. In addition, expression was monostable. These results indicate that FliZ is necessary for robust, bistable activation of SPI-1 gene expression by acetate and yeast extract. Nonetheless, there is still a small increase in the absence of *fliZ.* Possibly, this increase is due to growth enhancement by addition of yeast extract.

We also measured *hilA* promoter activity in a Δ*flhDC* mutant during growth in TB (Additional file 1: Fig. S11). Similar to what was observed with the Δ*fliZ* mutant, yeast extract in the presence of acetate was no longer able to induce bistable expression of the SPI-1 genes. In addition, acetate and yeast extract yielded a small, 2.5-fold increase in mean *hilA* expression. These results are again consistent with the hypothesis that the response to yeast extract is mediated by crosstalk between the flagellar and SPI-1 systems.

**Fig. 6:**
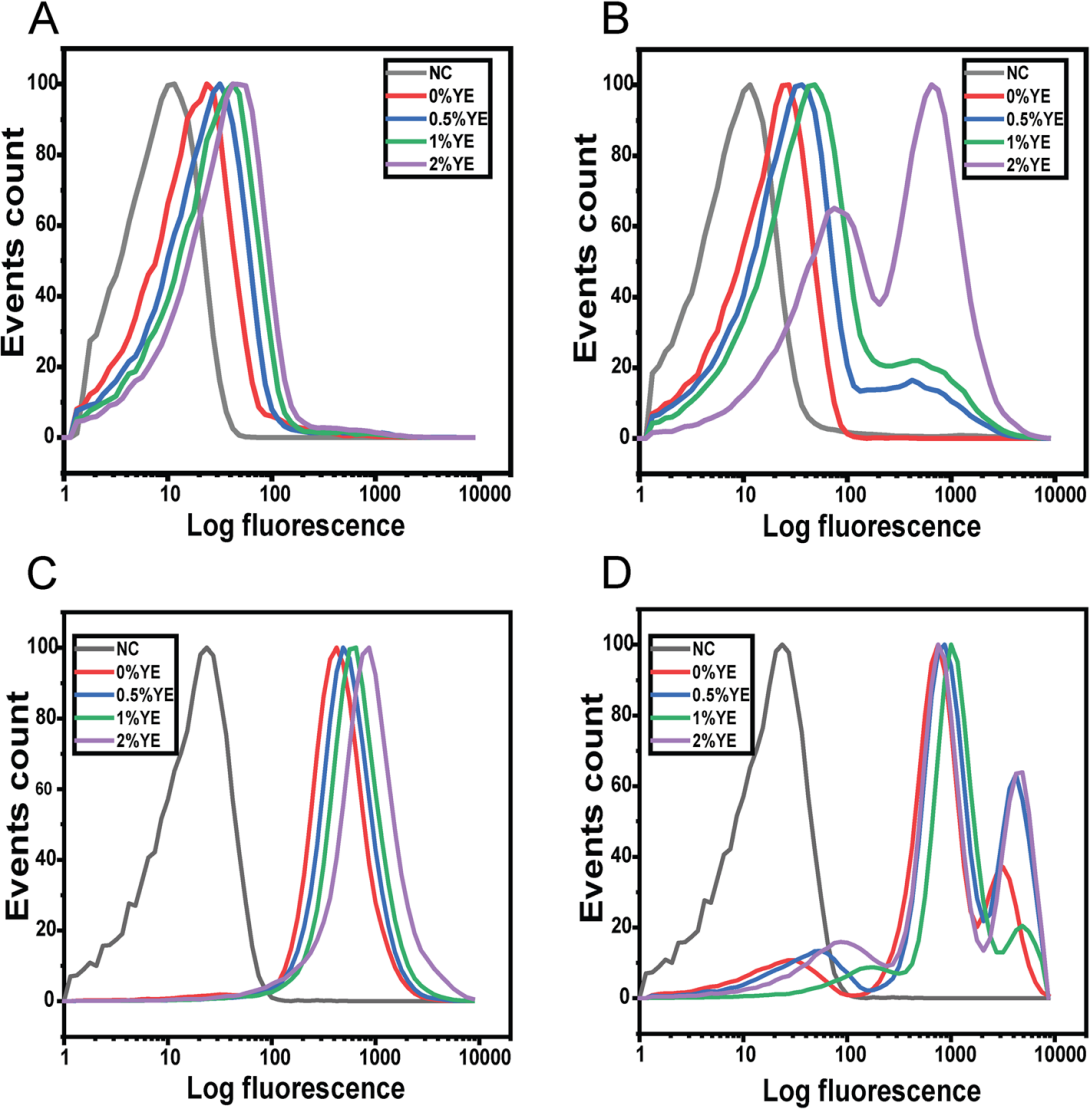
Response of the *hilA* promoter to acetate and yeast extract in a Δ*ydiV* mutant. Expression from *hilA* promoter was determined using single-copy transcriptional fusions to GFP as determined using flow cytometry. **a.** *hilA* promoter activity in the wild type during growth in TB medium at various concentrations of yeast extract (YE). **b.** *hilA* promoter activity in the wild type during growth in TB medium containing 10 mM acetate at various concentrations of yeast extract (YE). **c**. *hilA* promoter activity in a Δ*ydiV* mutant during growth in TB medium at various concentrations of yeast extract (YE). **d**. *hilA* promoter activity in a Δ*ydiV* mutant during growth in TB medium containing 10 mM acetate at various concentrations of yeast extract (YE). Negative control (NC) is the measured fluorescence of wild-type cells not containing the *gfp* gene during growth in TB medium. Panels **a** and **b** are shown for comparative purposes and are the same results as shown in Fig. 2

To better gauge the degree of crosstalk, we measured *hilA* promoter activity in a Δ*ydiV* mutant, again during growth in TB medium (Fig. 6). Loss of *ydiV* is known to strongly induces flagellar gene expression [30], and thus is expected to induce SPI-1 gene expression. Consistent with this hypothesis, *hilA* expression was induced in a Δ*ydiV* mutant during growth in TB medium, even in the absence of yeast extract (Fig. 6c). In addition, the addition of yeast only led to a small, 2-fold increase in *hilA* expression. These results are expected, because yeast extract represses the expression of YdiV. Interestingly, in the presence of acetate, we observed three populations of cells: one population where the SPI-1 genes were weakly expressed; a second where the SPI-1 genes were expressed at an intermediate level, similar to that observed in the absence of acetate; and a third population where the SPI-1 genes were highly expressed (Fig. 6d). In addition, only small changes in *hilA* expression were observed at different concentrations of yeast extract.

### Acetate represses flagellar gene expression

**Fig. 7:**
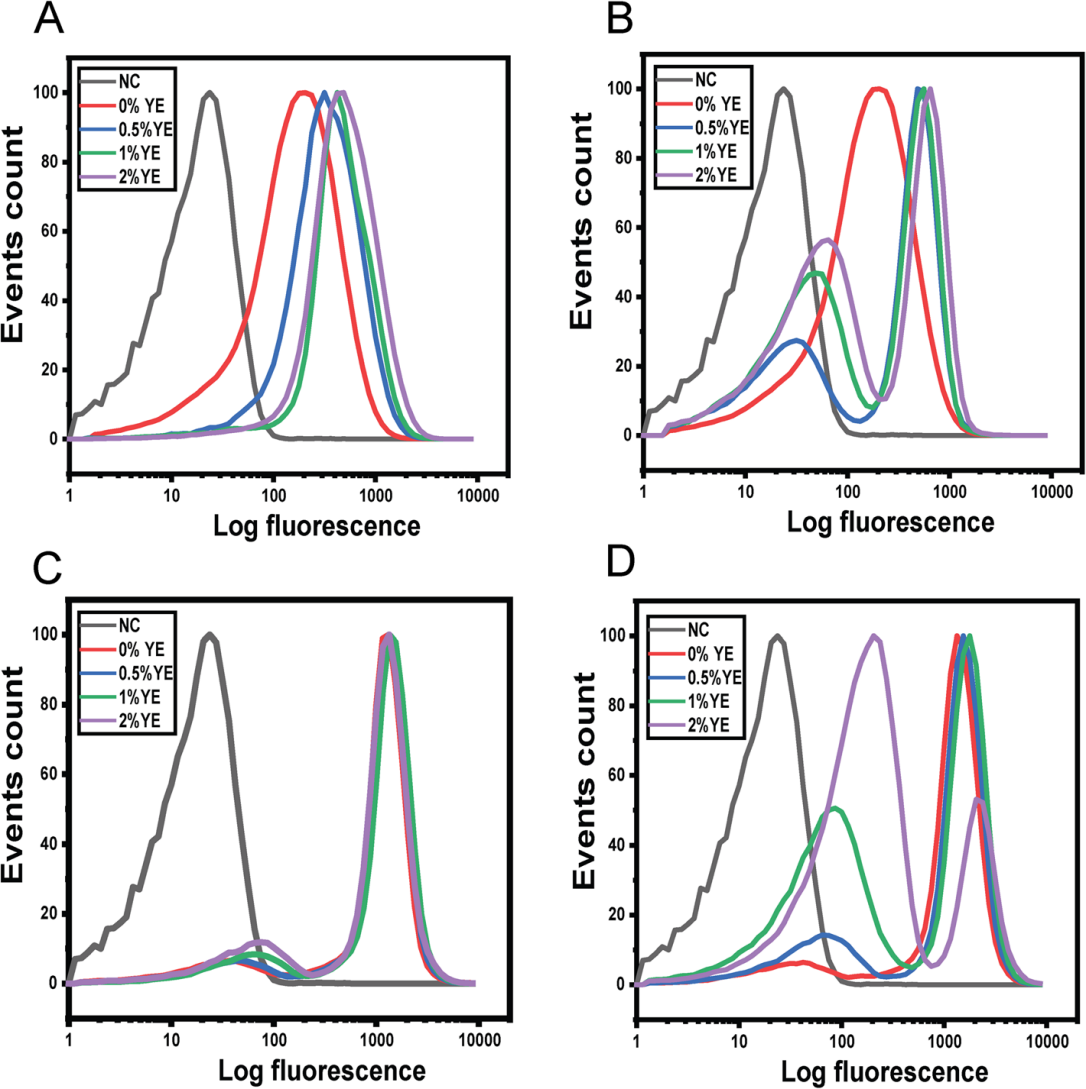
Acetate inhibits the expression of the flagella genes. Expression from *fliC* promoter was determined using single-copy transcriptional fusions to Venus as determined using flow cytometry. *fliC* promoter activity in wild-type cells during growth in TB medium (**a**) and growth in TB medium containing 10 mM sodium acetate (**b**) at various concentrations of yeast extract. *fliC* promoter activity in a Δ*ydiV* mutant during growth in TB medium (**c**) and growth in TB medium containing 10 mM sodium acetate (**d**) at various concentrations of yeast extract. Negative control (NC) is the measured fluorescence of wild-type cells not containing the *Venus* gene during growth in TB medium

To better understand the origins of the three populations, we also measured *fliC* promoter activity in the wild type and a Δ*ydiV* mutant during growth in TB in the presence of acetate and varying concentrations of yeast extract (Fig. 7). In the wild type, acetate does not affect *fliC* expression in the absence of yeast extract (Fig. 7a-b). However, the addition of yeast extract increase *fliC* expression in one population of cells and reduces it another. In addition, yeast extract increases the number of cells with reduced *fliC* expression*.* In the Δ*ydiV* mutant, the flagellar genes are strongly expressed in this mutant during growth in TB medium (Fig. 7c)**.** As expected, yeast extract does not affect expression. However, in the presence of acetate, yeast extract inhibits *fliC* expression (Fig. 7d). In particular, fewer cells strongly express *fliC* as the concentration of yeast extract increases, similar to what was observed in the wild type. In other words, yeast extract represses flagellar gene expression in a Δ*ydiV* mutant grown in the presence of acetate. Based on these results, trimodal expression of *hilA* in a Δ*ydiV* mutant likely results from the combination of two factors: one is the synergistic effect acetate and FliZ (through yeast extract) on *hilA* expression and the other is the reduction in FliZ expression by acetate in the presence of yeast extract. Since both effects occur in only a subset of cells, such a mechanism could explain the trimodal expression patterns seen in a Δ*ydiV* mutant (see Discussion for further details).

## Discussion

Both the flagellar and SPI-1 genes exhibit bistable expression patterns, with coexisting populations of cells expressing and not expressing these genes, depending on the growth medium. Previous work demonstrated that the concentration of yeast extract in the growth medium tunes the fraction of cells expressing the flagellar genes within the population (i.e. fraction of motile cells) during growth in VB medium [30]. In the present study, we explored whether yeast extract also tunes SPI-1 gene expression. The motivation for these experiments was prior work showing that flagellar and SPI-1 gene expressions are coupled. In particular, we hypothesized that yeast extract would induce SPI-1 gene expression through this crosstalk. To our initial surprise, we found that yeast extract had no effect on SPI-1 gene expression when added to VB medium; the same medium where yeast extract was previously shown to induce flagellar gene expression. Subsequent experiments using TB as the base medium also yielded negative results. However, when we added sodium acetate to the base growth medium, then yeast extract was capable of inducing SPI-1 gene expression. These results demonstrate that acetate and yeast extract act synergistically to induce SPI-1 gene expression.

Acetate is known to regulate SPI-1 gene expression through the action of the Bar/SirA two-component system [36]. The BarA/SirA two-component system post-transcriptionally regulates HilD expression through the action of CsrA, which binds to *hilD* mRNA and inhibits translation [39]. Yeast extract, on the other hand, increases the expression of the flagellar regulator FliZ by repressing the expression of YdiV, a negative regulator of flagellar gene expression. FliZ, in turn, increases HilD activity, post-translationally, by an unknown mechanism [14]. Based on our results, neither one of these systems alone is able to induce HilD expression under the growth conditions explored. Why then are both required for activation? Previous work has shown HilD, the master regulator of the SPI-1 gene network, positively regulates its own expression. This positive feedback loop amplifies HilD expression [5]. Our data would suggest that insufficient *hilD* mRNA is being produced during growth in TB or VB medium for acetate, through the action of the Bar/SirA two-component system, to induce HilD translation. Likewise, there is insufficient HilD for yeast extract, through the action of FliZ, to have any effect. However, when both signals are present, not only is HilD translation increased but also its activity. The combined effect of these two processes is likely sufficient to trigger the HilD positive feedback loop, leading to robust SPI-1 gene expression.

Such a mechanism would also explain the bistable expression patterns, where two populations of cells are observed. In one population, the positive feedback loop is not triggered and the cells do not express the SPI-1 genes. In the other population, the feedback loop is triggered and the cells express the SPI-1 genes. We note that these bistable expression patterns are only observed during growth in TB and not during growth in VB medium, a result that we cannot at this time explain. Nonetheless, the general mechanism is likely the same, because both signals are required for SPI-1 expression. The only difference is the manner by which HilD is expressed. Interestingly, flagellar gene expression is monostable during growth in TB medium and bistable during growth in VB medium.

One surprising observation concerns the expression patterns observed in a Δ*ydiV* mutant, where the flagellar genes are strongly expressed. In this mutant, the SPI-1 genes are expressed in all of the cells during growth in TB, even in the absence of yeast extract and acetate. These results would suggest that FliZ alone, when strongly expressed, is capable of inducing SPI-1 gene expression. In addition, we found that yeast extract had a small (2.5-fold) effect on SPI-1 gene expression in the absence of acetate, which is consistent with yeast extract primarily regulating SPI-1 gene expression through YdiV. However, in the present of acetate, two new populations emerge: one where the SPI-1 genes are weakly expressed and another where they are strongly expressed (> 200-fold increase). If we consider TB medium as the baseline, then the addition of acetate is expected to further increase SPI-1 gene expression, at least in a subpopulation of cells. This mechanism would explain the intermediate and high expression state. However, acetate also reduces flagellar gene expression in a subpopulation of cells, which in turn would reduce SPI-1 gene expression. This mechanism could potentially explain the low state.

Acetate is believed to repress flagellar gene expression because CsrA is known to post-transcriptionally induce *flhDC* expression [40–42]. In support of this hypothesis, we found that acetate reduces the numbers of cells strongly expressing the flagellar genes in both the wild type and in a Δ*ydiV* mutant. Interestingly, acetate strongly reduced these numbers in a yeast extract dependent manner. Most likely, this behavior results from acetate fermentation. During growth in yeast extract, the cells produce acetate (data not shown). Thus, more acetate is produced when cells are grown on higher concentrations of yeast extract, which further represses flagellar gene expression. Our data also suggest that this repression becomes significant with regards to SPI-1 gene expression only when the flagellar genes are strongly expressed, as it the case with a Δ*ydiV* mutant. Regardless of whether these results for the Δ*ydiV* mutant are physiologically significant, they nonetheless illustrate the complexity of the SPI-1 gene expression.

We note that yeast extract enhances the rate of growth in TB medium and acetate reduces it (Additional file 1: Fig. S12). In these regards, some of the differences in expression may be explained by differences in the rate of growth. However, growth rate alone does not explain the response of SPI-1 gene expression to acetate and yeast extract, as both can be eliminated by deleting the respective regulators. Thus, we cannot completely eliminate the effect of difference in growth rate; nonetheless, they are minor factors with regards to the observed responses.

A related question concerns the dynamics of SPI-1 and flagellar gene expression and the role of crosstalk in affecting the timing of gene expression within individual cells. Both the SPI-1 and flagellar gene networks exhibit complex temporal dynamics (e.g. [9, 32, 43–45]). However, we did not explore temporal dynamics in the present study. Nor is it clear how prior work translates to the condition explored in the present study. Further work will be required to answer these questions.

With regards to physiology, acetate is the most abundant short-chain fatty acid present in the distal small intestine, the site of invasion by *S. enterica*, with concentrations ranging from 10 to 30 mM [36]. Therefore, it is not surprising that 10 mM acetate induces SPI-1 gene expression, because it mimics the environment of small intestine. Yeast extract, on the other hand, is a complex mixture of nutrients. The specific factors within yeast extract that induce flagellar gene expression are not known [30], and the signal may simply be nutrients in general. In these regards, yeast extract may function as surrogate for other nutrients present in the small intestine. In addition, multiple other signals (e.g. pH and osmolarity) are known to induce the expression of the SPI-1 genes [35]. Our results demonstrate that *S. enterica* processes these signals in a nonlinear manner in order to control the relative fraction of cells expressing the SPI-1 invasion genes.

## Conclusions

We investigated the signals inducing the expression of the SPI-1 genes in *S. enterica*. We found that acetate and yeast extract were capable of synergistically activating SPI-1 gene expression. In addition, these signals were found to tune the fraction of cells expressing the SPI-1 genes. The governing mechanism involves transcriptional crosstalk with the flagellar gene network. Collectively, these results further our understanding of SPI-1 gene regulation and provide the basis for future studies investigating this complex regulatory mechanism.

## Methods

### Media

All experiments were performed in either tryptone broth (TB) (10 g/l tryptone) or Vogel-Bonner (VB) medium E (200 mg/l MgSO_4_.7H_2_O, 2 g/l citric acid monohydrate, 10 g/l anhydrous K_2_HPO_4_ and 3.5 g NaNH_4_PO_4_) [46] supplemented with 0.2% (w/w) glucose. Lysogeny broth (LB) (10 g/l tryptone, 5 g/l yeast extract, 10 g/l NaCl) was used for strain and plasmid construction. All media were buffered with 100 mM MOPS and the pH was adjusted to 7. Antibiotics were used at the following concentrations: ampicillin at 100 μg/μl, chloramphenicol at 20 μg/ml and kanamycin at 40 μg/ml.

### Strains

All *Salmonella* strains used in this study are isogenic derivatives of *Salmonella enterica* serovar Typhimurium 14028 (American Type Culture Collection). All gene deletions were made using the method of Datsenko and Wanner [47]. Single-copy transcriptional fusions to the flagella or SPI-1 promoters were used by cloning the promoter of interest upstream of either the green fluorescent protein or Venus and then integrating the plasmids into the chromosome using the CRIM method, as described previously [9, 30, 48]. The integrated plasmids were then moved into the wild type and the different mutants by P22 transduction. All the strains used in this study are listed in (Additional file 1: Table S1).

### Single-cell gene expression assay

Flow cytometry was used to measure fluorescence as a proxy for gene expression at single-cell resolution as described previously [48]. For the flagellar genes, cells were grown overnight at 37 °C in either TB medium or VB medium supplemented with 0.2% glucose. The overnight cultures were then diluted in to fresh medium (composition specified by the experiment) a starting OD600 of 0.02 and incubated with shaking at 37 °C to an OD600 of approximately 0.15. For the SPI-1 genes, the cells were first grown overnight in either TB medium or VB medium supplemented with 0.2% glucose under vigorous shaking (250 rpm) at 37 °C (SPI-1 repressing conditions), then sub-cultured to an OD of 0.05 into fresh medium (composition specified by the experiment), and grown statically (without shaking) in test tubes at 37 °C for 12 h; thus inducing a transition from SPI1-repressing to SPI1-inducing conditions [9].

Samples were collected and centrifuged at 3200×g for 10 min and resuspended in DAPI staining buffer with 14.3 μM DAPI and 50 μg/ml chloramphenicol. The cells were then incubated at room temperature for half an hour. All flow cytometry experiments were performed using a BD LRS II system from BD Biosciences. In all experiments, fluorescence values for approximately 50,000 events were recorded. Fluorescence values were measured using the Pacific Blue channel (excitation: 405 nm; emission: 450/50 nm) for DAPI and the FITC channel (excitation: 488 nm; emission: 530/30 nm) for the green fluorescent protein and Venus. Data extraction and analysis for the FACS experiments was done using FCS Express Version 6 (De Novo Software). The data were exported to Microsoft Excel (2016) and further processed to obtain the data for fluorescence and relative density distributions.

## Additional file

Additional file 1: **Table S1.** List of strains used in this study. **Figure S1.** Yeast extract weakly induce SPI-1 genes expression during growth in VB medium. **Figure S2.** Mean expression of hilA promoter during growth in VB medium. **Figure S3.** Comparison of flagellar gene expression during growth in TB and VB medium. **Figure S4.** Mean expression of fliC promoter during growth in TB and VB medium. **Figure S5.** Activation of SPI-1 gene expression by acetate and yeast extract during growth in TB and VB medium. **Figure S6.** Mean expression of hilA promoter during growth in VB medium with or without 10 mM acetate. **Figure S7.** Activation of hilD promoter by acetate and yeast extract during growth in TB medium. **Figure S8.** Mean expression of hilD promoter during growth in TB medium with or without 10 mM sodium acetate. **Figure S9.** Mean expression of hilA promoter in a ΔsirA mutant. **Figure S10.** Mean expression of hilA promoter in a ΔfliZ mutant during growth in TB medium with or without 10 mM sodium acetate. **Figure S11.** Response of the hilA promoter to yeast extract is due to transcriptional crosstalk with the flagellar system as determined using a ΔflhDC mutant. **Figure S12.** Comparison of growth in TB in the presence or absence of acetate and yeast extract. (DOCX 9960 kb)

## Data Availability

The datasets used and/or analysed during the current study are available from the corresponding author on reasonable request.
