# Supplementary material for: Synergistic action of SPI-1 gene expression in Salmonella enterica serovar typhimurium through transcriptional crosstalk with the flagellar system
Source: BMC Microbiol. 2019 Sep 5;19:211. doi: 10.1186/s12866-019-1583-7 (PMC6727558; doi:10.1186/s12866-019-1583-7)
Supplement: Supplementary file 1 — Table S1. List of strains used in this study. Figure S1. Yeast extract weakly induce SPI-1 genes expression during growth in VB medium. Figure S2. Mean expression of hilA promoter during growth in VB medium. Figure S3. Comparison of flagellar gene expression during growth in TB and VB medium. Figure S4. Mean expression of fliC promoter during growth in TB and VB medium. Figure S5. Activation of SPI-1 gene expression by acetate and yeast extract during growth in TB and VB medium. Figure S6. Mean expression of hilA promoter during growth in VB medium with or without 10 mM acetate. Figure S7. Activation of hilD promoter by acetate and yeast extract during growth in TB medium. Figure S8. Mean expression of hilD promoter during growth in TB medium with or without 10 mM sodium acetate. Figure S9. Mean expression of hilA promoter in a ΔsirA mutant. Figure S10. Mean expression of hilA promoter in a ΔfliZ mutant during growth in TB medium with or without 10 mM sodium acetate. Figure S11. Response of the hilA promoter to yeast extract is due to transcriptional crosstalk with the flagellar system as determined using a ΔflhDC mutant. Figure S12. Comparison of growth in TB in the presence or absence of acetate and yeast extract. (DOCX 9960 kb) [file 12866_2019_1583_MOESM1_ESM.docx]

**SUPPLEMENTAL DATA**

| Strain | Relevant characteristics |
| --- | --- |
| 14028 | Wild type, serovar *Typhimurium* |
| SH02 | *attλ::P_hilD_-*Venus |
| SH24 | *ΔflhDC attλ::P_hilA_-gfp* |
| SH52 | *attλ::P_hilA_-gfp* |
| SH25 | *ΔfliZ attλ::P_hilA_-gfp* |
| SK405 | attλ:: *P_fliC_*-Venus |
| SH54 | Δ*ydiV* attλ::*P_hilA_-gfp* |
| SH56 | Δ*sirA* attλ:: *P_hilA_-gfp* |
| SK407 | Δ*ydiV* attλ:: *P_fliC_*-Venus |

**Table S1.** List of strains used in this study.


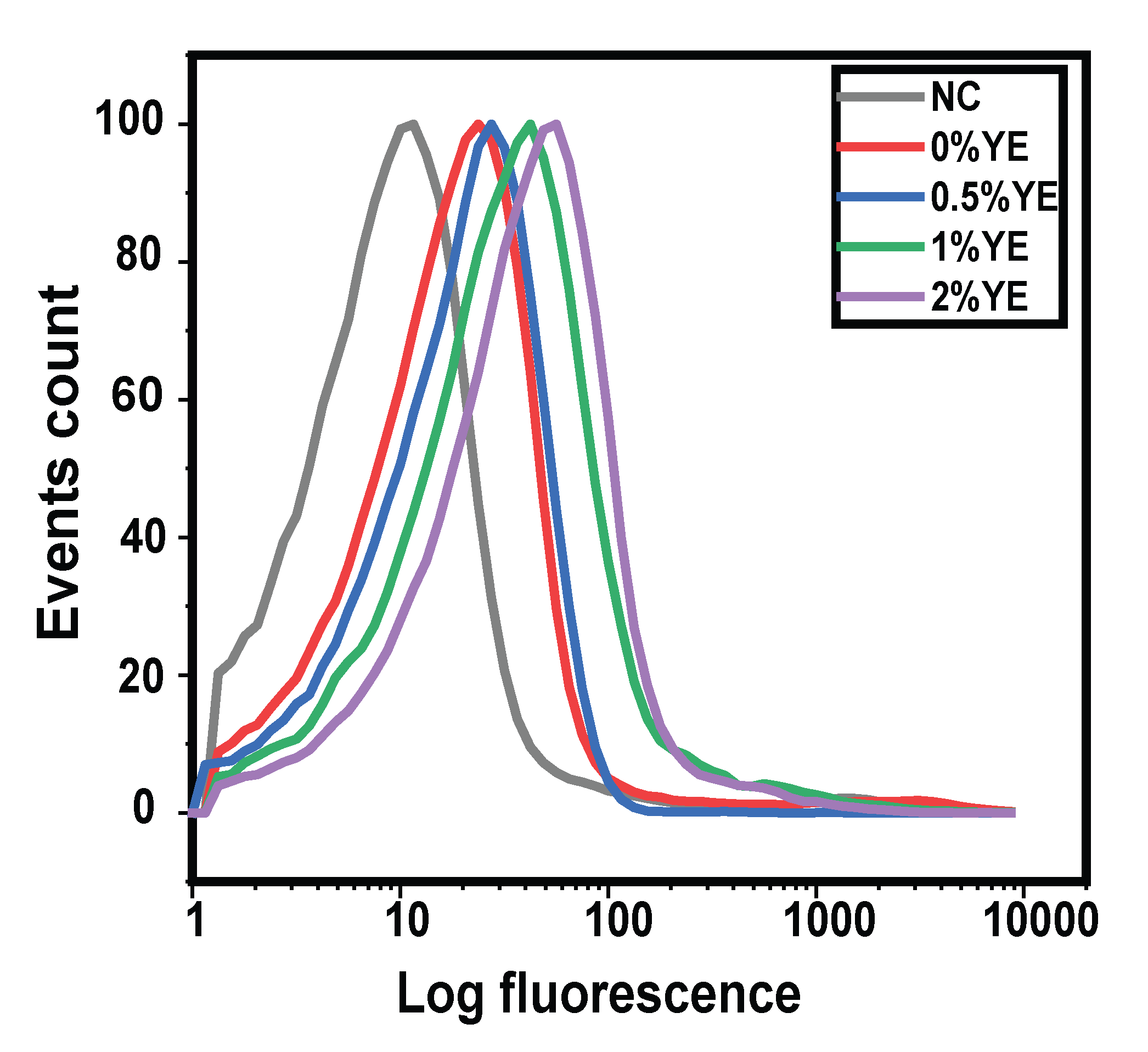


**Figure S1**. Yeast extract weakly induce SPI-1 genes expression during growth in VB medium. Expression from *hilA* promoter was determined using single-copy transcriptional fusions to GFP as determined using flow cytometry. Figure shows *hilA* promoter activity in wild-type cells during growth in VB medium at various concentrations of yeast extract. Negative control (NC) is the measured fluorescence of wild-type cells not containing the *gfp* gene during growth in VB medium. Analysis of data is provided in **Figure S2**.


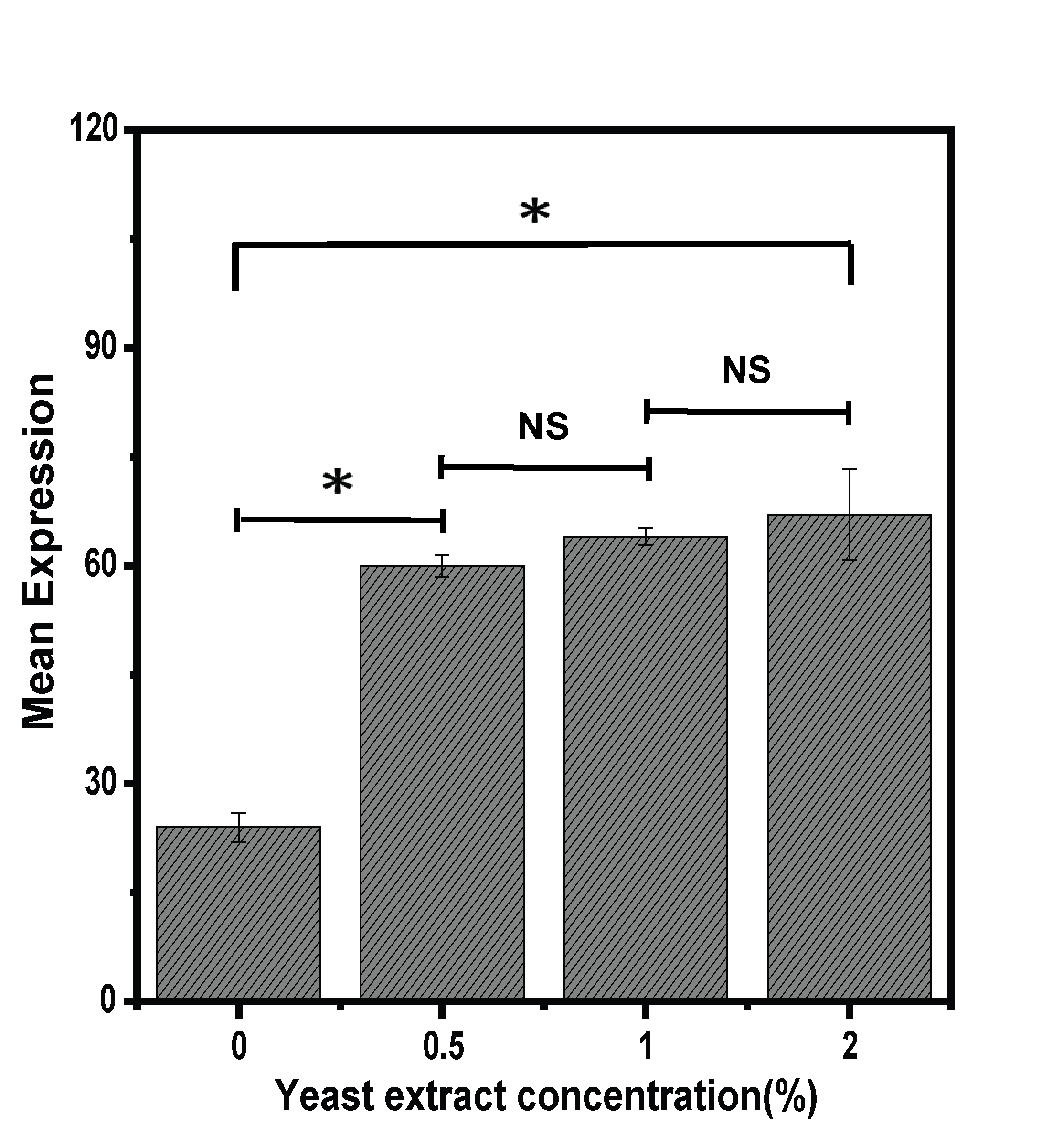


**Figure S2.** Mean expression of *hilA* promoter during growth in VB medium. Expression from *hilA* promoter was determined using single-copy transcriptional fusions to GFP as determined using flow cytometry. Representative data are provided in **Figure S1**. Error bars denote the standard deviation from three biological replicates. NS: not significant; *: p<0.05.

**
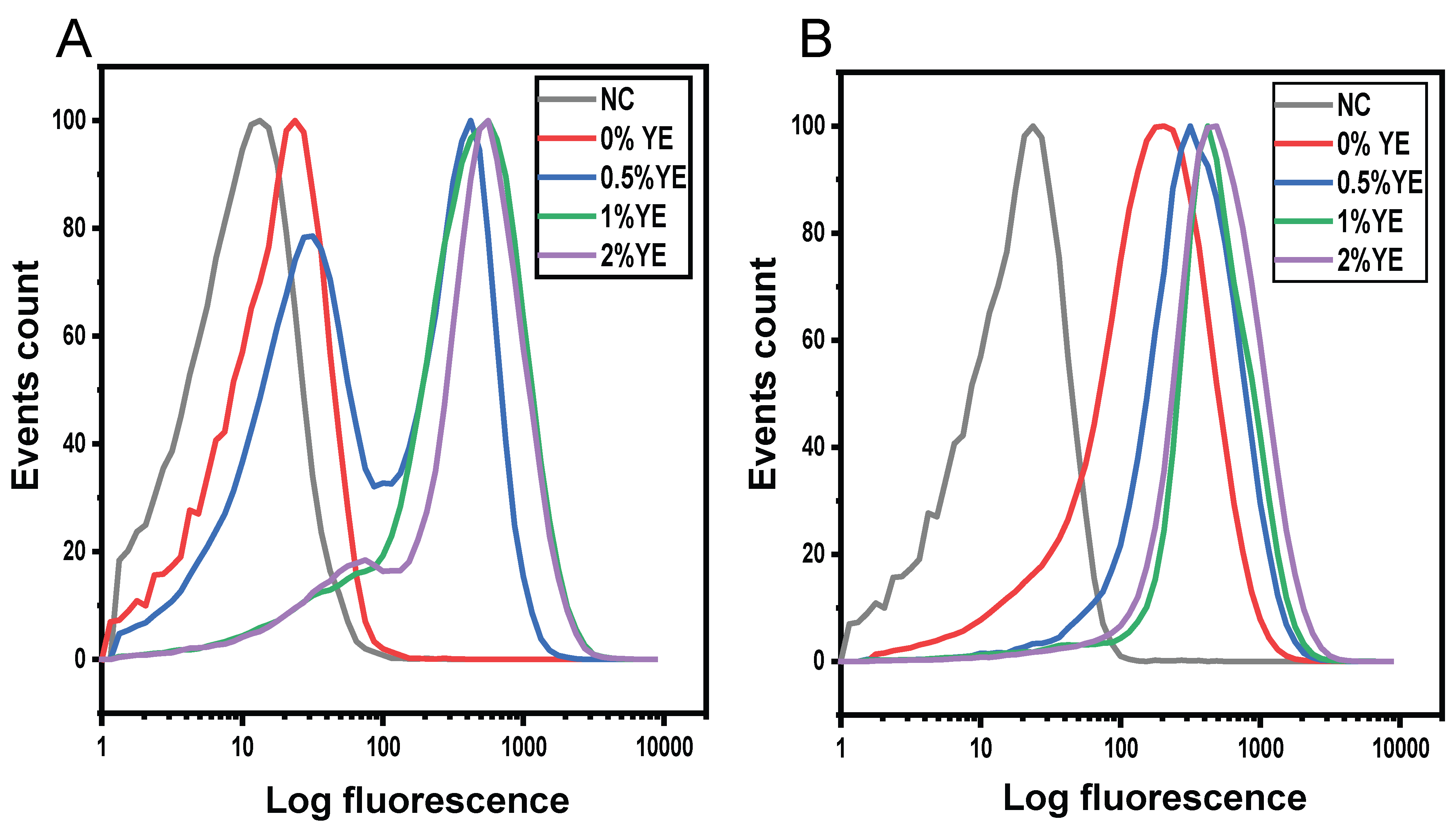
**

**Figure S3.** Comparison of flagellar gene expression during growth in TB and VB medium. Expression from *fliC* promoter was determined using single-copy transcriptional fusions to Venus as determined using flow cytometry. *fliC* promoter activity in wild-type cells during growth in VB medium (**A**) and growth in TB medium containing (**B**) at various concentrations of yeast extract. Negative control (NC) is the measured fluorescence of wild-type cells not containing the *gfp* gene during growth in TB medium. Analysis of data is provided in **Figure S2**.


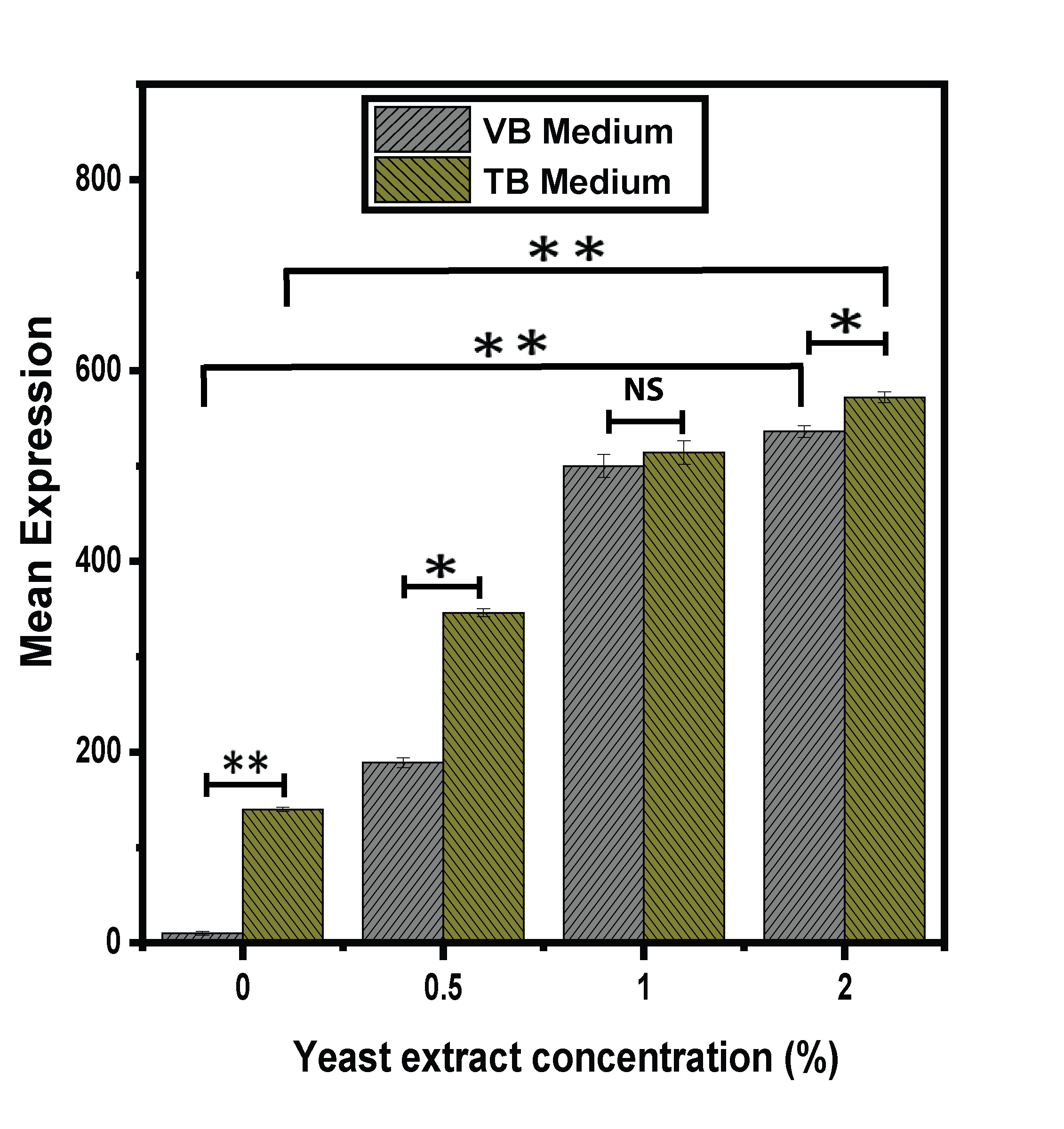


**Figure S4.** Mean expression of *fliC* promoter during growth in TB and VB medium. Expression from *fliC* promoter was determined using single-copy transcriptional fusions to Venus as determined using flow cytometry. Representative data are provided in **Figure S3**. Error bars denote the standard deviation from three biological replicates. Error bars denote the standard deviation from three biological replicates. NS: not significant; *: p<0.05; **: p<0.001.


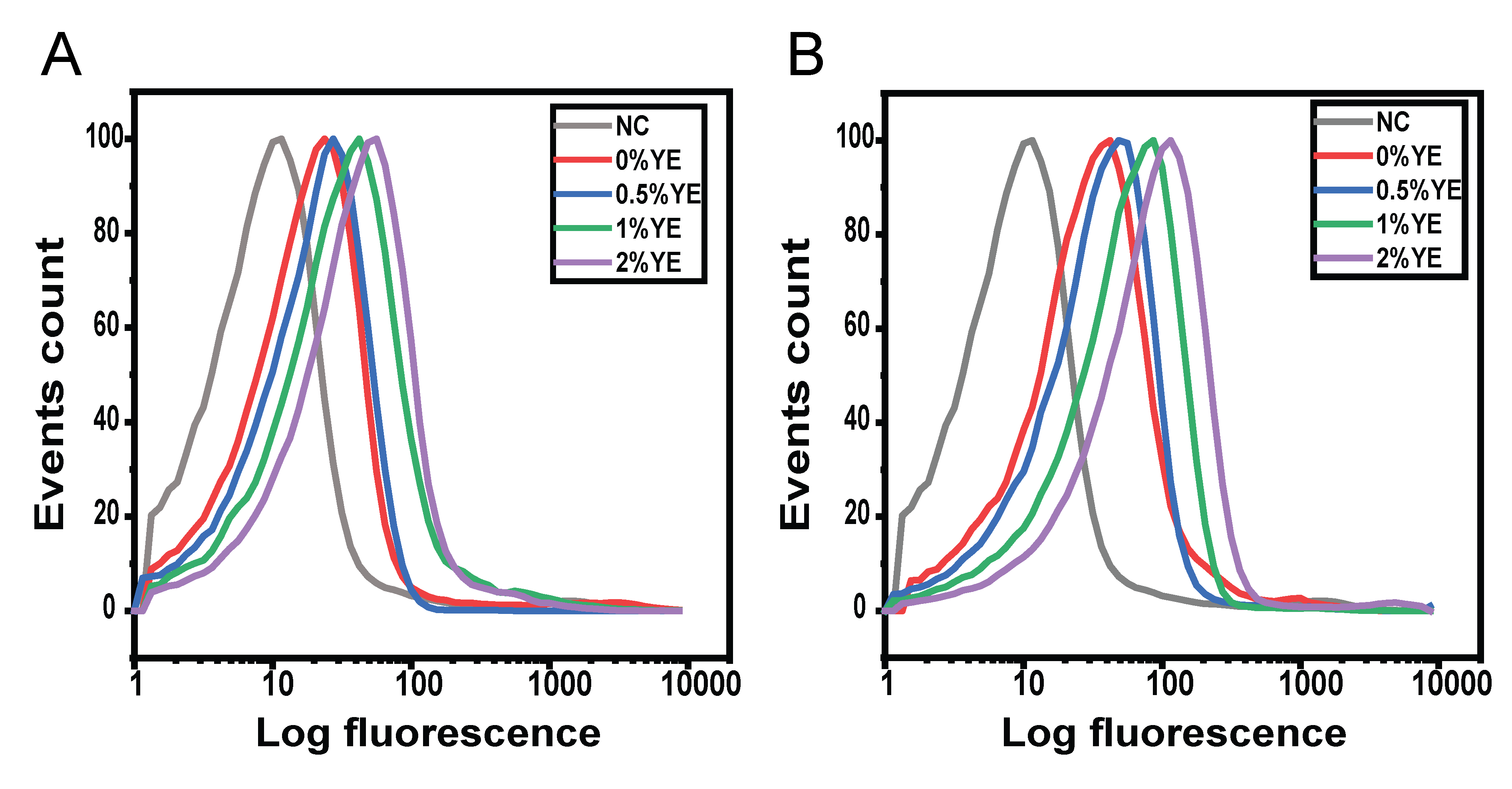


**Figure S5.** Activation of SPI-1 gene expression by acetate and yeast extract during growth in TB and VB medium. Expression from *hilA* promoter was determined using single-copy transcriptional fusions to GFP as determined using flow cytometry. *hilA* promoter activity in wild-type cells during: growth in VB medium (**A**) and growth in VB medium containing 10 mM sodium acetate (**B**) at various concentrations of yeast extract. Negative control (NC) is the measured fluorescence of wild-type cells not containing the *Venus* gene during growth in VB medium. Analysis of data is provided in **Figure S6**.


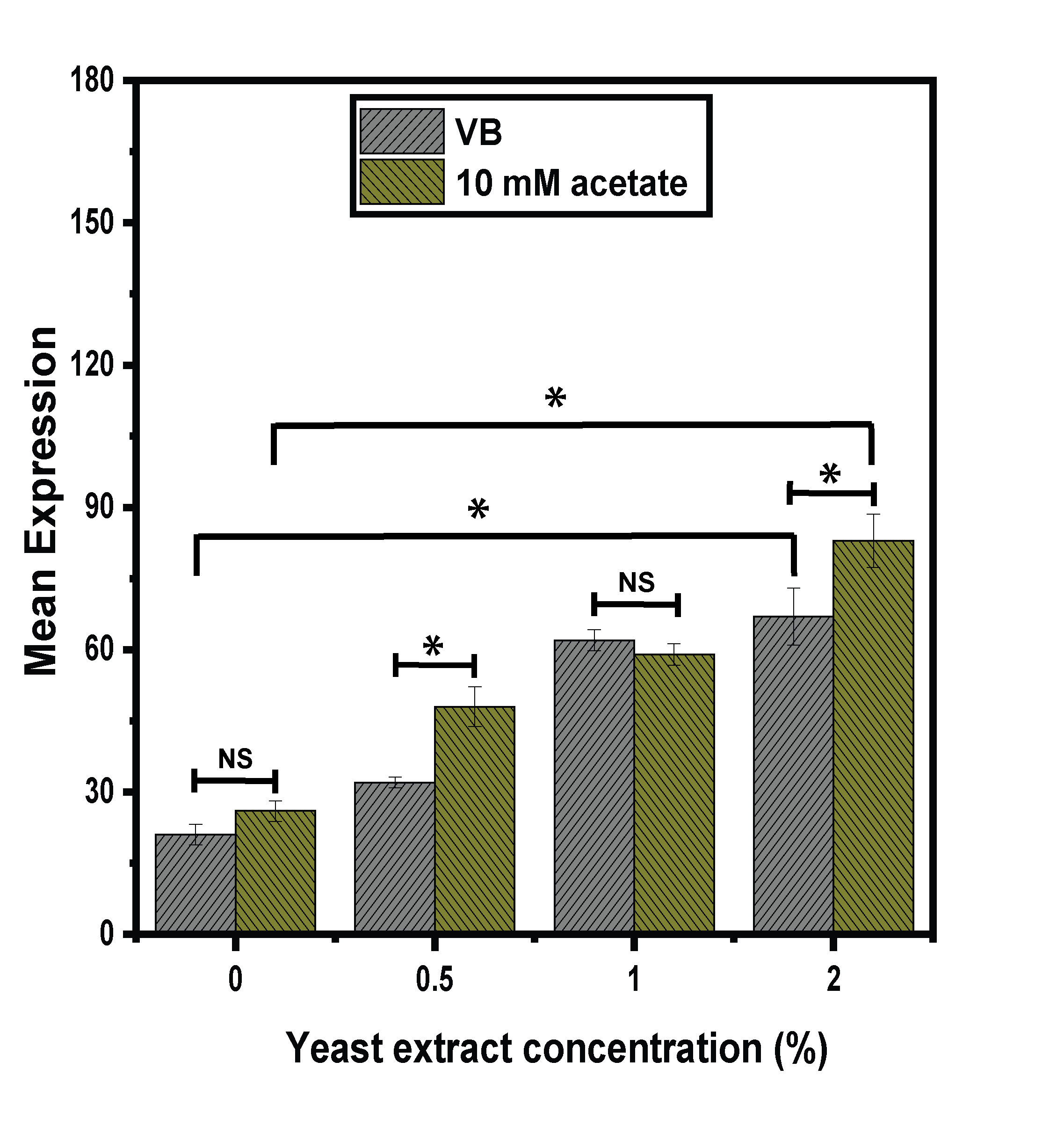


**Figure S6.** Mean expression of *hilA* promoter during growth in VB medium with or without 10 mM acetate. Expression from *hilA* promoter was determined using single-copy transcriptional fusions to GFP as determined using flow cytometry. Representative data are provided in **Figure S5**. Error bars denote the standard deviation from three biological replicates. NS: not significant; *: p<0.05.


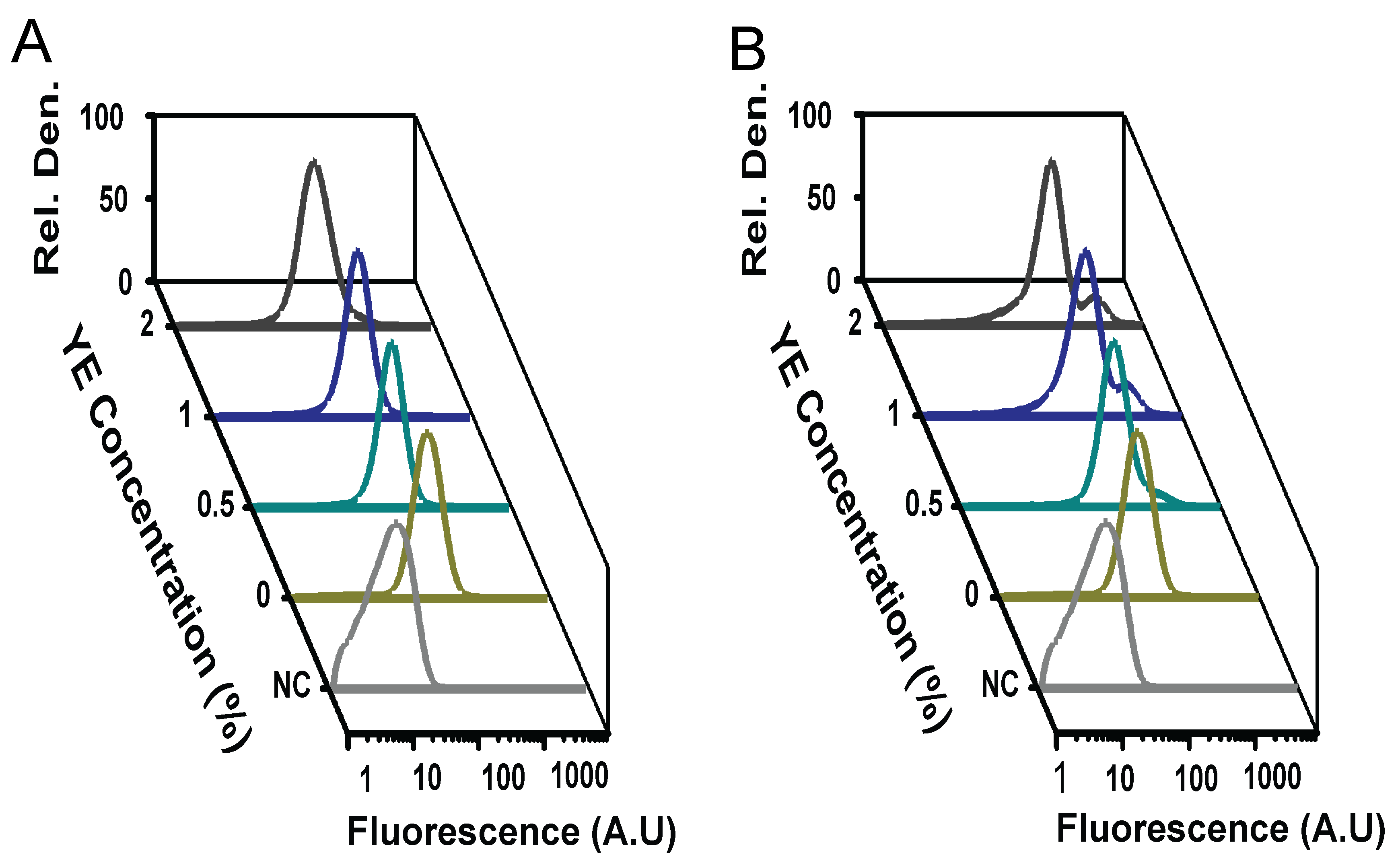


**Figure S7.** Activation of *hilD* promoter by acetate and yeast extract during growth in TB medium. Expression from *hilD* promoter was determined using single-copy transcriptional fusions to Venus as determined using flow cytometry. *hilD* promoter activity in wild-type cells during: growth in TB medium (**A**) and growth in TB medium containing 10 mM sodium acetate (**B)** at various concentrations of yeast extract. Negative control (NC) is the measured fluorescence of wild-type cells not containing the *gfp* gene during growth in TB medium. Analysis of data is provided in **Figure S8**.


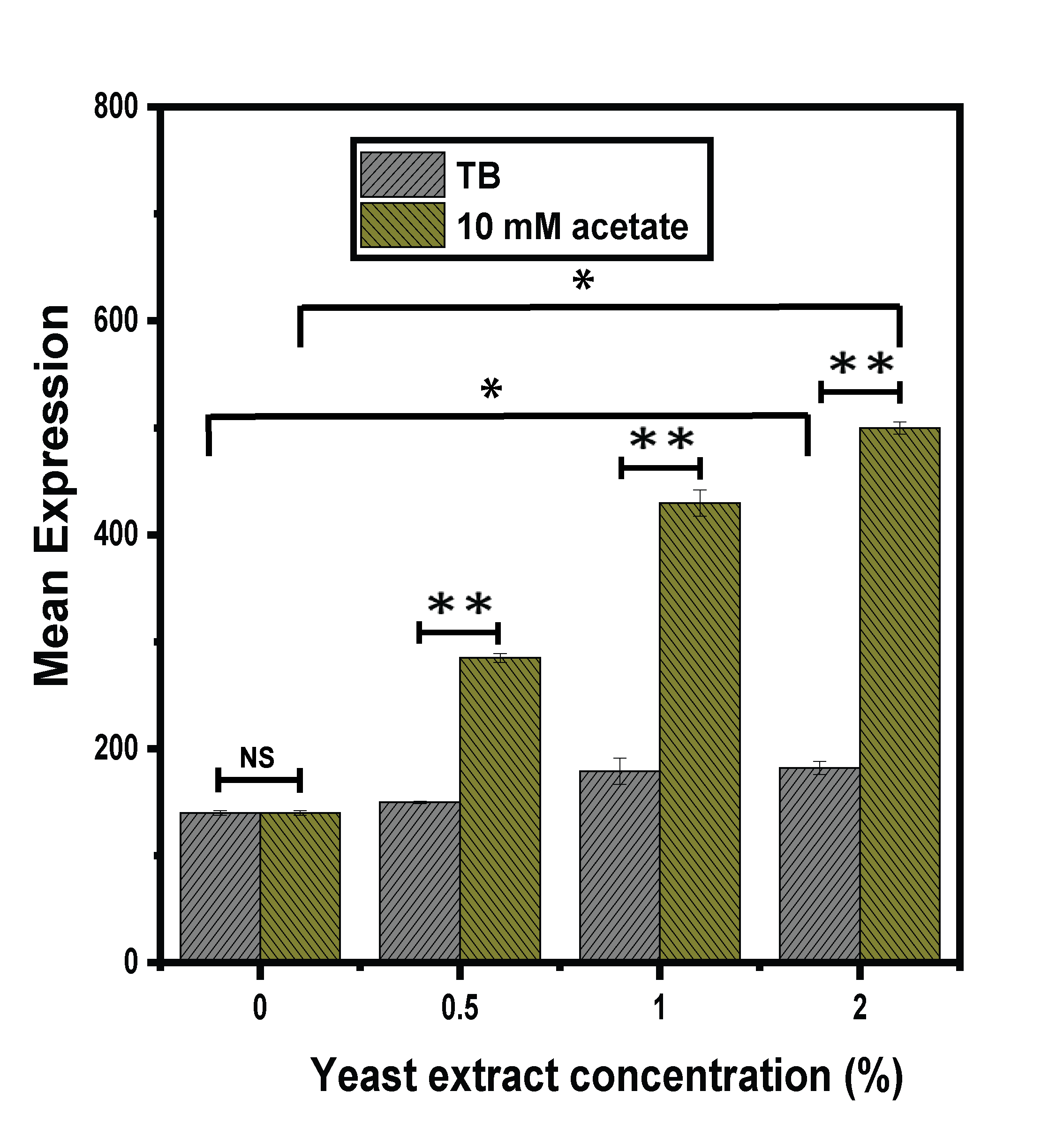


**Figure S8.** Mean expression of *hilD* promoter during growth in TB medium with or without 10 mM sodium acetate. Expression from *hilD* promoter was determined using single-copy transcriptional fusions to Venus as determined using flow cytometry. Representative data are provided in **Figure S7**. Error bars denote the standard deviation from three biological replicates. NS: not significant; *: p<0.05; **: p<0.001.


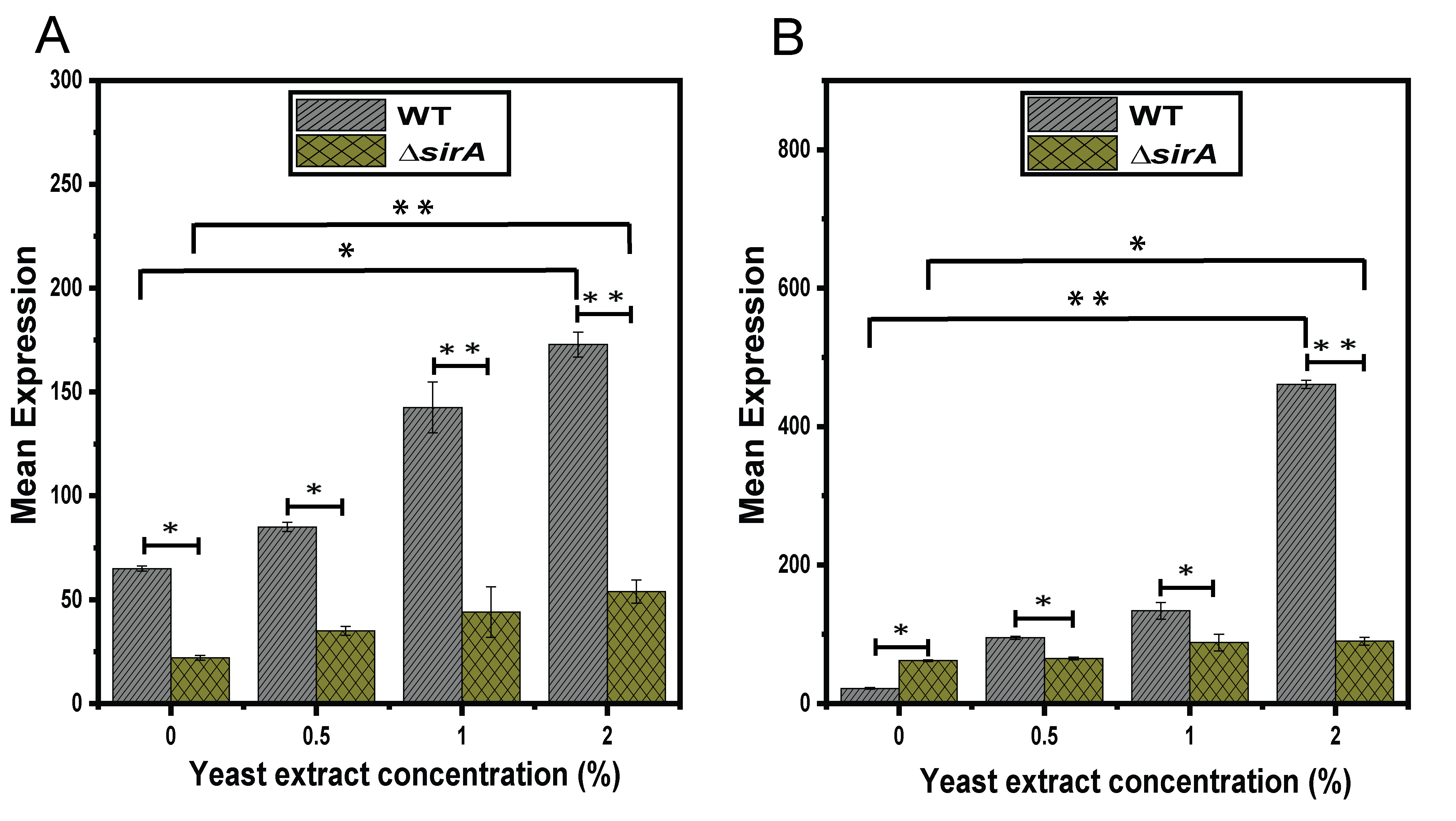


**Figure S9.** Mean expression of *hilA* promoter in a ∆*sirA* mutant. Panel (A) compares the expression between wild type and ∆*sirA* mutant during growth TB. Panel (B) compares the expression between wild type and ∆*sirA* mutant during growth in presence of 10 mM sodium acetate. Expression from *hilA* promoter was determined using single-copy transcriptional fusions to Venus as determined using flow cytometry. Representative data are provided in **Figure 4**. Error bars denote the standard deviation from three biological replicates. NS: not significant; *: p<0.05; **: p<0.001.


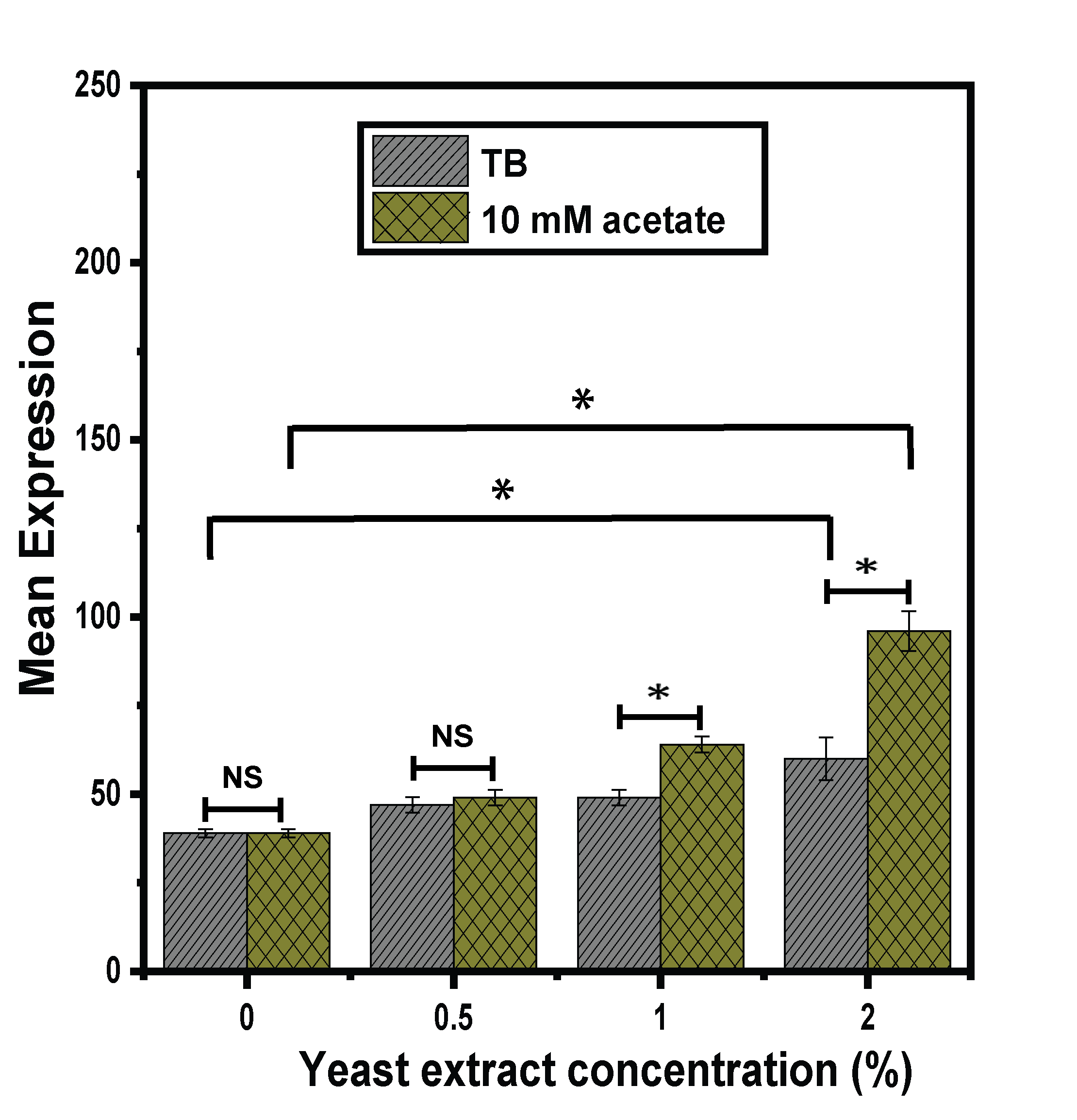


**Figure S10.** Mean expression of *hilA* promoter in a ∆*fliZ* mutant during growth in TB medium with or without 10 mM sodium acetate. Expression from *hilA* promoter was determined using single-copy transcriptional fusions to Venus as determined using flow cytometry. Representative data are provided in **Figure 5.** Error bars denote the standard deviation from three biological replicates. NS: not significant; *: p<0.05; **: p<0.001.


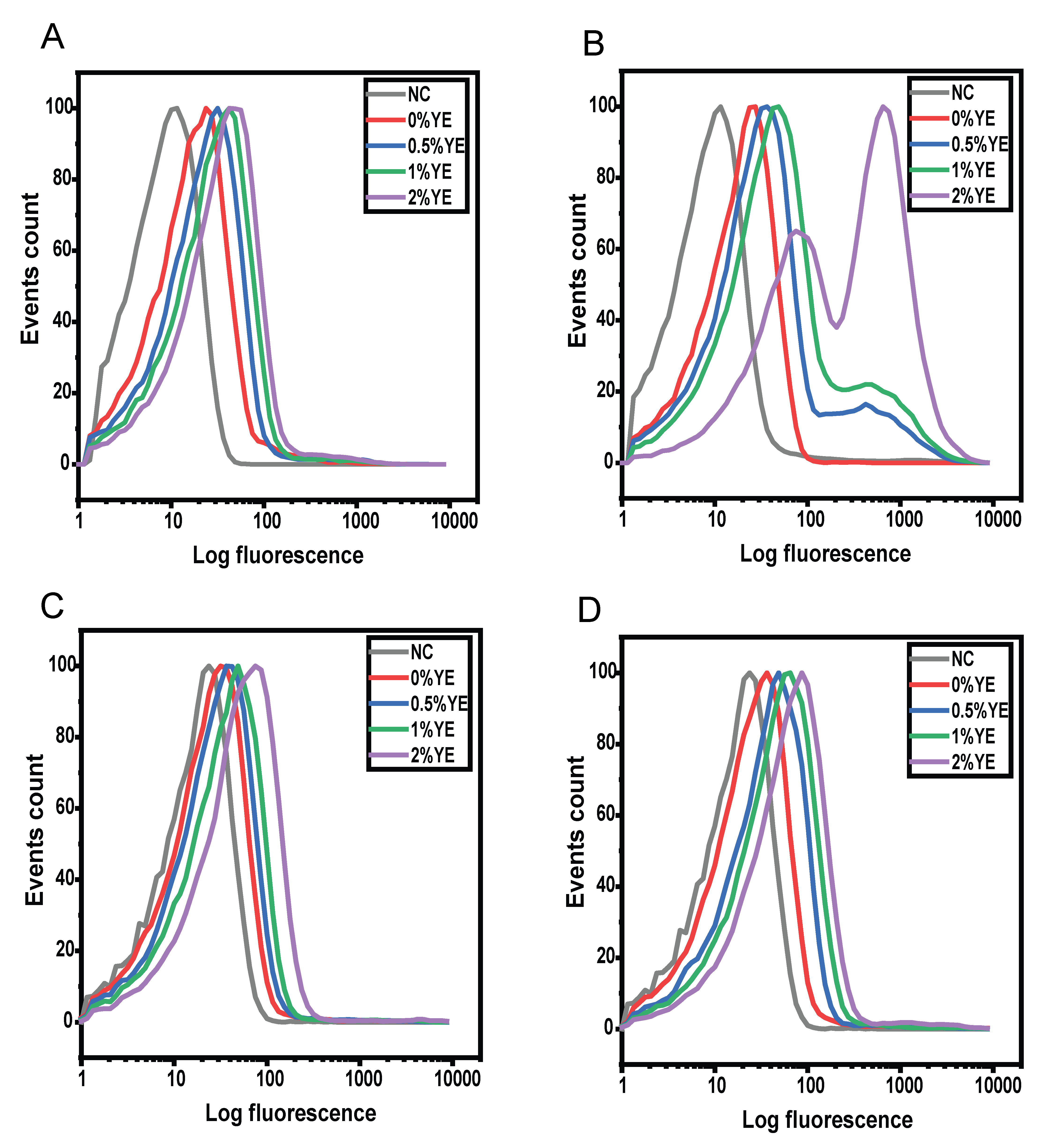


**Figure S11**. Response of the *hilA* promoter to yeast extract is due to transcriptional crosstalk with the flagellar system as determined using a Δ*flhDC* mutant. Expression from *hilA* promoter was determined using single-copy transcriptional fusions to GFP as determined using flow cytometry. **A.** *hilA* promoter activity in the wild type during growth in TB medium at various concentrations of yeast extract (YE). **B**. *hilA* promoter activity in the wild type during growth in TB medium containing 10 mM acetate at various concentrations of yeast extract (YE). **C.** *hilA* promoter activity in a Δ*flhDC* mutant during growth in TB medium at various concentrations of yeast extract (YE). **D**. *hilA* promoter activity in a Δ*flhDC* mutant during growth in TB medium containing 10 mM acetate at various concentrations of yeast extract. Negative control (NC) is the measured fluorescence of wild-type cells not containing the *gfp* gene during growth in TB medium. Panels A and B are shown for comparative purposes and are the same results as shown in **Figure 2**.


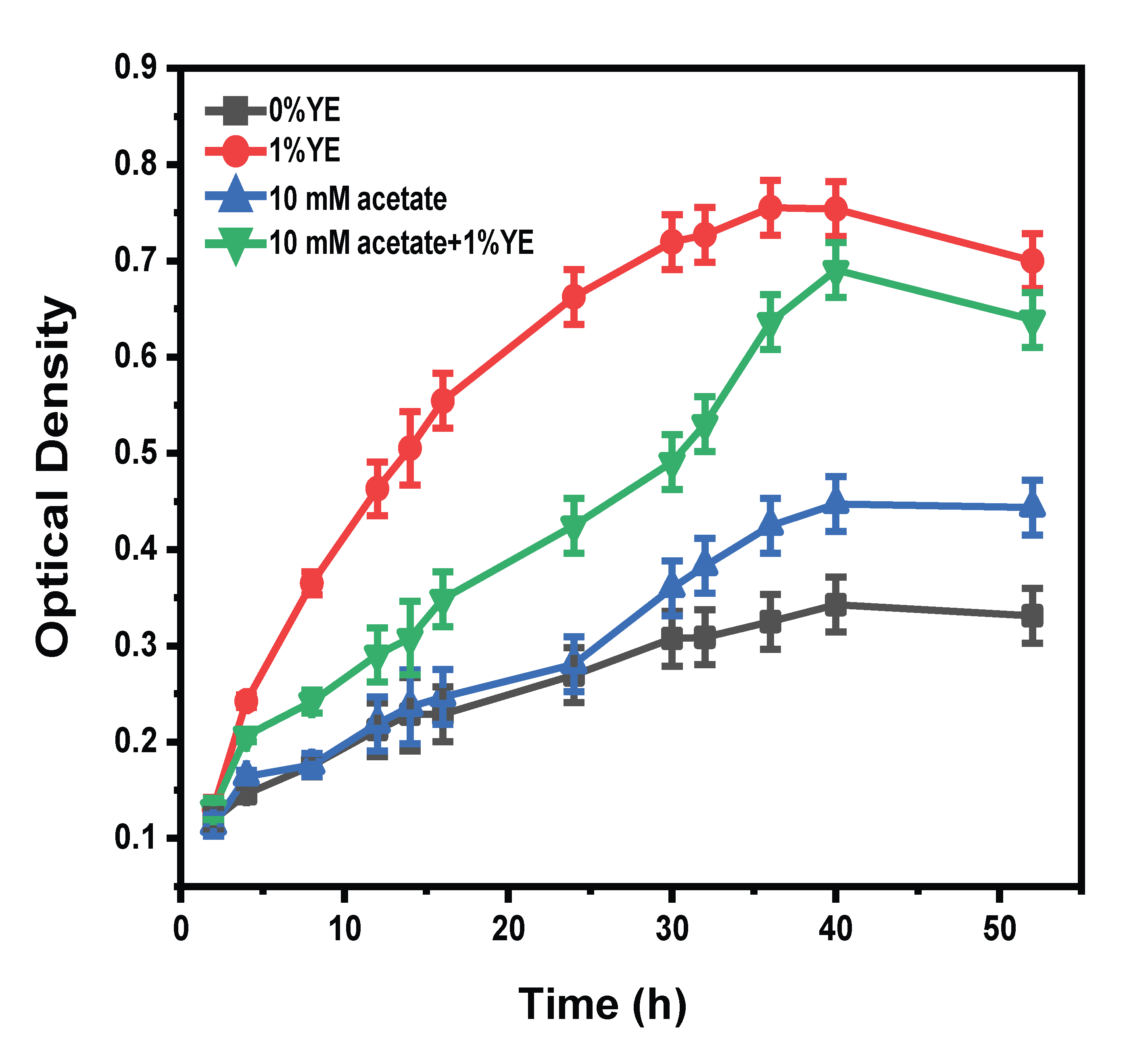


**Figure S12.** Comparison of growth in TB in the presence or absence of acetate and yeast extract. Cells were grown statically in test tubes. Error bars denote standard deviations from three biological replicates.
